# Obsessive-Compulsive Disorder During the COVID-19 Pandemic Process: A Narrative Review

**DOI:** 10.5152/eurasianjmed.2022.22221

**Published:** 2022-12-01

**Authors:** Fatma Tuygar Okutucu, Hacer Akgul Ceyhun

**Affiliations:** 1 Department of Psychiatry, Atatürk University Faculty of Medicine, Erzurum, Turkey

**Keywords:** COVID-19, contamination, obsessive-compulsive symptoms, liability for harm, adults

## Abstract

This study aimed to review all empirical contributions published between March 2020 and June 2022, addressing the impact of the COVID-19 pandemic on obsessive-compulsive disorder in adults. We searched the literature in PubMed, Google Scholar, and Web of Science. A total of 543 articles were scanned and 73 full-text articles were identified. Reviews, comments, letters to the editor, and case reports (except case series) were excluded. It was determined that 42 articles met the inclusion criteria, 6 studies involving only children and adolescents were excluded, and 36 were decided on their suitability for our study. The analysis determined that COVID-19 had an impact on obsessive-compulsive disorder. Characteristics such as liability for harm and unacceptable thoughts influenced the symptoms as well as contamination and washing. Some studies showed an increase in the severity of obsessive-compulsive symptoms, while some reported no worsening but improvement with treatment and follow-up. While some reported variations in outcomes related to sociodemographic characteristics and subtypes, some focused on the risk of suicide. There were also studies conducted on special groups such as pregnant women or healthcare workers. The results were controversial. While available data contain more information on worsening obsessive-compulsive disorder symptoms, data on the status of patients under treatment were scarce. There were not enough studies evaluating follow-up results. Influencing factors such as sociodemographic characteristics, subtypes, comorbid conditions, treatment, and support did not seem to have been adequately addressed.

**Main Points**

- People with obsessive-compulsive disorder (OCD) were vulnerable to the psychological effect of the COVID-19 pandemic and many studies have been conducted on this subject.
- Studies reported more information on worsening OCD symptoms.
- Studies evaluating follow-up and treatment results were few.
- There is a need for the development of the studies. More studies are essential to capitalize on more appropriate and early interventions and to understand how variables exacerbate OC symptoms in the OCD population.

## Introduction

After the World Health Organization declared the coronavirus (COVID-19) outbreak a pandemic in March 2020^1^ dramatic measures were taken, such as quarantining cities with thousands of people to limit the spread of the virus. The effect of measures such as social distance, self-isolation, and quarantine on vulnerable individuals with mental health problems has been an important research topic for scientists.

An important issue of mental health that may be affected by the pandemic is obsessive-compulsive symptoms. Obsessive-compulsive disorder (OCD) symptoms are characterized by intrusive thoughts (obsessions) and repetitive behaviors (compulsions) aimed to reduce the anxiety of the obsessions.^2^ Its prevalence in the general population is 2%-3%.^3^

The common contact of OCD symptoms with epidemic measures is contamination concerns and washing/cleaning compulsions.^4^ There is a significant overlap between contamination/washing symptoms of OCD and recommended public health recommendations (hand washing, using face masks, wiping around, etc.) to prevent the spread of COVID-19.^5^ Other OCD themes include obsessions about COVID-19 (intrusive thoughts about the growing number of COVID-19 cases), hoarding (over-supply of food and other needs), and controlling (exaggerated and repetitive monitoring of the news about coronavirus) behaviors.^6^

The health risk and anxiety of the COVID-19 pandemic may have a special impact on individuals susceptible to OCD, by both worsening the existing symptoms and accelerating new symptoms.^6^ However, the fear of contamination provides preventive behaviors for COVID-19,^7^ and in a way, OCD symptoms also play a protective role.^8^

In OCD, concerns about COVID-19 may be considered to have responsibility for being infected or having undetected symptoms, as well as infecting and harming someone else.^6^ Another impact of the pandemic on OCD symptoms is that people with OCD may tend to attach personal meaning to viruses and microbes. As these features involve rituals such as increased handwashing, they can lead to prolonged distress and anxiety during the COVID-19 outbreak.^9^ For individuals with OCD, the pandemic can also be a process of providing exposure and response prevention therapy.^10,11^

The aim of our review was to explore the latest literature on the effect of the COVID-19 pandemic on OCD patients and identify the limitations. Particularly, we wanted to assess whether OCD symptoms were worsening and which issues of OCD were particularly affected. Our main purpose was to analyze and synthesize all published empirical studies from March 2020 to June 2022 that had identified the impact of COVID-19 pandemic on OCD.

### Research Question

The aim of the present narrative review was to evaluate existing contributions by researching the impact of coronavirus pandemic on OCD in adults and highlighting their limitations. We especially wanted to verify if there had been a worsening of OCD symptoms and which OCD subtypes were mostly affected. Our main objective was to analyze all studies from March 2020 to June 2022 concerning OCD and the coronavirus pandemic focusing on clinical populations of adults and analyze the current literature. We suggest that research on OCD in times of pandemics is important because the effect of such a global situation might be continued and a pandemic might be repeated.

## Methods

We analyzed all studies from March 2020 to June 2022 concerning OCD and the coronavirus pandemic focusing on clinical populations of adults. The aim of this review was to evaluate existing contributions by researching the impact of coronavirus pandemic on OCD in adults. We included original studies investigating the impact of the COVID-19 pandemic on OCD and obtained information from studies focusing on different countries.

The literature search was conducted using the following databases: PubMed, Google Scholar, and Web of Science. Key words searched for our results were “COVID-19,” “coronavirus,” “pandemic,” “contamination,” “OCD,” “obsessive-compulsive symptoms,” “adults,” and “harm liability” which were used in different combinations.

### Eligibility Criteria

The studies with the following criteria were included: peer-reviewed academic journals published between March 2020 and June 2022; a clinical sample of OCD in an adult population and empirical study on the impact of the COVID-19 pandemic; cross-sectional or longitudinal study design; and accessible abstracts and articles with full texts.

Studies with the following criteria were excluded: not making an original contribution (e.g., review, comment, case report, or letter to the editor). Typology of treatment, presence of comorbidity, publication status, and language and nationality of the contributor were not exclusion criteria.

### Search Strategy

**Figure 1.**
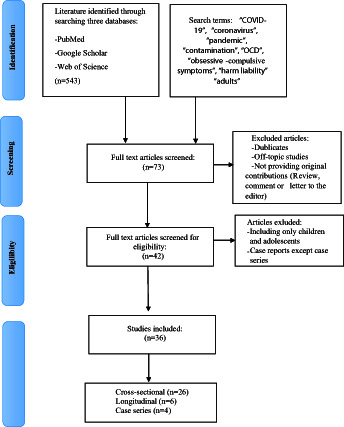
Flowchart on search strategy.

A total of 543 articles were scanned and 73 full-text articles were identified. Reviews, comments, letters to the editor, and case reports (except case series) were excluded. It was determined that 42 articles met the inclusion criteria, 6 studies involving only children and adolescents were excluded, and 36 were decided on their suitability for our study (Figure 1).

## Results

The characteristics of reviewed articles are illustrated in Table 1.

### Analysis of Findings

#### Study Characteristics

The studies showed 6 different research designs: 26 cross-sectional studies, 2 also included qualitative assessments, 4 case–control studies, 1 description, 6 longitudinal studies, and 1 with case series reporting. Research designs are illustrated in Table 1.

The results were provided by quantitative–qualitative evaluations (questionnaires, semi-structured interviews, self-reports, and online evaluations),^12-34^ face-to-face interviews,^35-44^ or telephone calls.^20,23,36,45^ In 1 study, data were obtained from the opinion of clinicians.^25^ Conclusion criteria are illustrated in Table 1.

As of February-March 2020, studies were conducted in many countries during the first curfew period and in the following periods. One study compared the results from 2019 with other data collected during the first quarantine (January-May 2020).^35^

#### Sample Characteristics

In general, the sample groups evaluated in the studies were diverse in terms of gender, age, and comorbidity. Studies included both small and larger-scale trials (Table 1).

Two studies had 2 samples of groups with and without a diagnosis of OCD.^28,46^ In 2 studies conducted on pregnant women, the status of pregnant and non-pregnant women was compared.^42^ Another study was a comparison between health workers working in the COVID-19 service and in different services.^43^ In addition, Jelinek et al^20^ evaluated 2 OCD subsamples, washers, and non-washers to compare compulsions during the COVID-19 pandemic. There was only 1 study evaluating OCD-related disorders such as hoarding disorder (HD) and skin-picking disorder (SPD).^19^ Tandt et al^40^ study was the only study that evaluated the status of families as well as OCD patients.^40^ Obsessive-compulsive disorder patients were the participants who were with different comorbidities such as anxiety disorder,^18,22,23,26,30,46^ or mixt anxiety disorder and depressive disorder.^17,24,27,28,31,33,41,43^ In some studies, comorbid diagnoses were excluded.^19,40^ In other studies, psychiatric comorbidity was not specified.^21,25,29,39,41,45,47^

##### Obsessive-Compulsive Symptoms During the Pandemic in Individuals with a Pre-Pandemic Diagnosis of Obsessive-Compulsive Disorder

There were 16 studies evaluating the impact of the COVID-19 pandemic process on individuals previously diagnosed with OCD.^12,14,16,24,25,28,35,36,37,38,39,40,41,44,45,46,^ Wheaton et al^12^ reported an increase in OCD symptoms as well as an increase in anxiety about COVID-19, with 58.3% of respondents reporting that anxiety about COVID-19 had become an obsession with OCD symptoms, while 41.7% conveyed anxiety about COVID-19 as a separate problem. Obsessive-compulsive disorder symptoms have been found to be associated with contamination and liability for harm. Davide et al^35^ evaluated the status of OCD patients during pandemic process who were in remission and those who were not in remission before the pandemic, and it was found that there was a significant increase in OCD symptoms and more worsening in those who were in remission before the pandemic. Tukel et al^44^ reported that OC symptom severity worsened in 60% of OCD patients during the pandemic. 

Two studies by Khosravani et al^36,37^ specifically found that all symptom dimensions of OCD were affected during the COVID-19 pandemic, not only contamination obsessions but also responsibility for harm and reported that situations such as responsibility for harm, stress response to trauma, and unacceptable intrusive thoughts were effective in the increasing risk of suicide. 

In 3 longitudinal studies that conducted treatment follow-up studies,^39,41,45^ improvement in OCD symptoms was reported in OCD patients followed up with acceptance and commitment therapy (ACT) and exposure response prevention (ERP) therapies. No significant symptomatic exacerbation was reported in the study in which the results of regular follow-ups of 2 months and 6 months with cognitive behavioral therapy (CBT), ERP, and pharmacological treatment were shared^41^ and in which pharmacological treatments were followed.^45^ In the study, in which clinicians’ opinions were evaluated and the results of follow-up with ERP were reported,^25^ the clinicians showed that the symptoms of 38% of their patients worsened during the pandemic and the symptoms did not change although 47% participated in ERP.

The study that evaluated the early and long-term effects of COVID-19 on OCD patients demonstrated that depressive, anxiety, and OCD symptoms worsened during early COVID-19 and the negative effect continued at 1-year follow-up.^24^ In 1 study, the coping attitudes of OCD patients were evaluated; positive reframing, acceptance, and humor were lower, while the results on instrumental support and religion were better in OCD group.^46^ The OCD group showed higher rates of denial and self-blame, and cognitive coping and spiritual coping were statistically significant. While the control group mostly used cognitive strategies, the OCD group mostly used emotional strategies. Study of Tandt et al^40^ also included families of patients and showed that the COVID-19 crisis increased OCD symptoms of almost all patients, decreased their ability to use coping strategies, and they needed increased family support. The pandemic had strained patients’ social and family lives and had led to withdrawal from face-to-face therapy, leaving many to feel less supported. The importance of including family support in the therapy process was emphasized.

### Obsessive-Compulsive Symptoms of the General Population During the Pandemic Process

The study by Zheng et al^13^ investigating the prevalence of OCD in an urban population in China showed the OCD prevalence as 17.96%. Aggression and contagion obsessions and compulsions to clean, check, and repeat were evident. Two studies found increased prevalence of OCD during COVID-19 pandemic among the general population.^32,33^ Another study that was conducted to examine the prevalence of OCD symptoms in pregnant women found the prevalence as 7.12% of participants, more than twice as high as rates of peripartum OCD reported prior to the pandemic.^34^

In Samuels et al’s study^15^ evaluating whether COVID-19 triggers OCD or not, 22% of the reported symptoms related to transmission were considered as contagious obsessions and 20% as contagion phobia. Loosen et al^17^ evaluated how the course of OCD, depression, anxiety symptoms, and the search for information and following guidelines about COVID-19 were affected by these symptoms during the first wave of the pandemic and found that OCD, depression, and anxiety were increased in the first wave of the pandemic, and depression decreased in the later wave, anxiety drew a straight line, OCD continued to increase, information-seeking behavior increased at the beginning of the pandemic and then decreased, and was especially found to be associated with OC symptoms. In a study that evaluated intrusive thoughts about COVID-19 and OCD in individuals without OCD,^18^ OCD and COVID-19 intrusive of those with high OCD symptoms were at a similar rate as those with low OCD symptoms. Severity of OCD symptoms was significantly correlated with the frequency of COVID intrusions and the amount of their distress. In the study, in which OCD subtypes (OCD-related disorders) were mentioned, an increase in subtypes such as HD, SPD, hair picking disorder, decreased quality of life, and increased disability rate were reported. In 2 studies conducted by Jelinek et al^20,21^ on the general population, worsening of OCD symptoms was reported. 

In the study by Taher et al^22^ in which OCD prevalence, related factors, and correlations were evaluated among medical students, 57% were evaluated as normal, while 43% as probable OCD requiring further evaluation. About 70% had accompanying psychiatric symptoms, anxiety, and stress. Unpleasant thoughts were the most common symptoms in 51.8%. The study by Yassa et al.^42^ on pregnant women showed more OCD and less anxiety symptoms compared to non-pregnant women. State anxiety levels did not differ between the trimesters. In a study conducted on healthcare professionals in Turkey,^43^ healthcare professionals working in COVID-19 services showed a significant increase in OCD, depression, and anxiety symptoms compared to healthcare professionals working in other services. There was only 1 study that evaluated the pre-pandemic period and compared the period before and after the pandemic and evaluated whether insomnia before COVID-19 would be a predictor for OCD symptoms.^47^ Having insomnia symptoms before the pandemic showed a small increase in OCD symptoms in the COVID-19 period. In the study by Al-Shatanawi et al.^26^ which evaluated self-reported obsessions for COVID-19 preventive measures among medical students, 6.8% of participants reported obsessions related to preventive measures against COVID-19, and 93.2% (n = 1308) showed that they had no obsessions.^26^ In a study evaluating the prevalence of OCD symptoms during the pandemic process, screening was conducted among university students in 3 different periods. In questionnaire 1, 11.3% of the participants were identified as probable OCD. In surveys 2 and 3, 3.6% and 3.5% of respondents had scores indicating probable OCD, respectively.^30^ In another study, the prevalence of OCD symptoms increased at a significantly higher rate than reported pre-pandemic rates for the sample population.^31^

### The Course of Symptoms Associated with the Contamination

There were few studies specifically evaluating OCD symptoms associated with contamination. Wheaton et al^12^ and Davide et al^35^ reported that an increase in OCD symptoms was evident in symptoms related to contamination. The prevalence study by Zheng et al^13^ reported that aggression and contagion obsessions and compulsions to clean, check, and repeat were prominent. The study by Khosravani et al^36^ reported an increase in all OCD symptoms and an increase in both contamination-related symptoms and obsessions such as responsibility for harm, symmetry, and unacceptable thoughts. Another study reported an increase in symptoms related to contamination by 64.7% and symptoms related to harm by 56.2%.^14^ A study found that some of the symptoms related to contamination were obsessions and some were phobias,^15^ and there were also results indicating that the dimensions of OCD symptoms were variable and that the obsessions were multiple.^16,38^ The study comparing washers and non-washers reported that OCD symptoms increased more in washers.^20^ In the study evaluating medical students, the contamination and washing subscales were found to be low-scored.^22^ In the study conducted on pregnant women, an increase in OCD symptoms and anxiety was reported and symptom subtype was not specified, but no correlation was found between anxiety and hygiene-cleaning subscales.^42^ There was also a study reporting that contamination symptoms increase anxiety, but it did not specifically report an increase in contamination symptoms.^27^ There were 6 studies reporting an increase in contamination, washing, and cleaning in particular.^12,13,15,20,35,36^ One study reported improvement with ACT and ERP therapies, suggesting that the pandemic process may be an opportunity to achieve overexposure and treatment goals.^39^ Another study reported that complying with washing and hygiene rules within the scope of COVID-19 measures did not increase washing compulsions.^45^

### Factors Associated with Obsessive Compulsive Symptoms

Studies that found the increased OC symptoms of people with OCD to be associated with COVID-19 anxiety,^12,18,23,26,29,40,43^ as well as studies that reported increased obsessive symptoms, especially related to the responsibility to do harm, were also present.^14,27,36,37^ In a study reporting that responsibility to harm was an important cause of the increase in OCD symptoms,^37^ it has been reported that responsibility to harm and unacceptable thoughts, as well as OCD severity, stress response to COVID-19, traumatic stress reactions, control compulsions, comorbid depression, and anxiety affect suicidal ideation. Another study reporting an increased risk of suicide reported that deterioration in obsessive symptoms was also associated with internet control for reassurance, the need for family support, and sleep disturbances.^15^ A study reported that symptomatic worsening was more common during the COVID-19 pandemic process in those who reported that they were in remission before quarantine,^35^ and another study that evaluated the pandemic process reported that sleep disorder before the pandemic had little effect on OCD.^47^ In the prevalence study conducted on the pregnant women, younger maternal age, income loss, and suspected SARS-CoV-2 infection were all associated with higher OC symptoms.^34^ The study that investigated how the pandemic has affected OCD patients found that COVID-19 obsession levels were correlated with anxiety severity.^44^

In a prevalence study conducted on the general population,^13^ being single, being a student, family history of OCD and other mental disorders, comorbid psychiatric comorbidity, and prolonged sleep latency were risk factors for OCD. In another prevalence study,^31^ higher OCD symptoms were evaluated to be associated with higher stress. 

Generalized anxiety disorder (GAD), and major depressive disorder (MDD). In a study investigating the prevalence of OCD among medical students,^22^ the presence of other psychological symptoms such as anxiety, depression, sleep disorders, eating disorders, and stress was significantly associated with probable OCD. In a study investigating the prevalence of OCD among university students,^30^ fear intensity was found to be positively associated with probable OCD rate and mean total scores for Yale-Brown Obsession Compulsion Scale (Y-BOCS), with a higher probability of OCD in non-medical fields.

In a study evaluating the coping strategies of OCD patients during the pandemic,^46^ it was reported that some positive strategies and maladaptive strategies differ in their use. In the OCD group, comorbidity influenced greater use of inappropriate strategies (denial, substance abuse, and self-blame). The subtype of obsessions-compulsions was not effective in their use. Anxiety and depression symptom severity was associated with more maladaptive behaviors.

A study evaluating the early and long-term effects of the pandemic on OCD patients demonstrated that female gender, concern about COVID-19, and baseline OCD level and exacerbation of OCD symptoms were risk factors, while optimism was a factor of mental toughness which is protective against increase of OCD both in early period and follow-up.^24^ In another study,^28^ the OCD group scored higher in perceived anxiety and depression levels, experienced suicidal thoughts more often, and experienced more frequent changes in perceived eating and sleep patterns. In a case–control study in pregnant women, an increase in OCD symptoms but a decrease in anxiety symptoms was reported in pregnant women, and it was stated that this situation may be related to the use of coping strategies.^42^

In the study conducted by Hojgaard^14^, a group of 29.4% reported that they accepted their obsessive thoughts more during the pandemic process. 

The study evaluating healthcare workers stated that the increase in OCD symptoms of healthcare workers working in the COVID-19-positive service was associated with COVID-19 anxiety.^43^

### Treatment Characteristics

Besides several studies showing an increase in OC symptoms in adults despite the possibility of psychopharmacological treatment and psychotherapy (CBT and/or ERP),^12,24,25,28,35,36,37^ there was also 1 study reporting that drug compliance was higher in patients with OC deterioration.^38^ Improvement was reported in OCD patients in the follow-up study by Kuckertz et al^24^ with ACT–ERP therapies and by Carmi et al.^41^ with CBT, ERP, and pharmacological treatment.

In the study by Chackraborty et al.^45^ exacerbation was reported in only 5-6 of 84 patients, and they were those who could not take their medications because they could not go to the pharmacy. In the study by Storch et al^41^ in which the ERP results of OCD patients during the pandemic were evaluated, it was stated that ERP progression was weak. In Alonso et al’s study,^28^ all participants were on pharmacological therapy, 25.1% of patients required a change in pharmacological therapy during the first months of the pandemic, and all except 2 were given increasing doses of selective serotonin reuptake inhibitors (SSRI) or clomipramine (n = 16), antipsychotic doses (n = 3), or received increased or added doses of benzodiazepines.

## Discussion

This study aimed to analyze and review the studies investigating the impact of the COVID-19 pandemic on OC symptoms in adults. The COVID-19 pandemic may cause worsening of pre-existing psychiatric illnesses. Especially, recent research has indicated that COVID-19 may have an impact particularly on people with OCD. It has been observed that COVID-19 is an important source of stress for individuals with OCD.^12,16,37^ In the early stages of the pandemic, OC symptoms increased both in those with OCD and in the general population.^13,15,20,33,34,39^ It can be thought that warnings about the coronavirus and reminders for hygiene have exacerbated obsessive concerns about contamination.^14,20,35^ The results showed that people with OCD had high concerns on the spread of pandemic diseases, including COVID-19. COVID-19 had become an important problem, especially in those with contamination obsessions and those with OCD who had more fear of COVID-19.^15,23,35,44^

Many studies had found a worsening of the symptom dimensions of OC symptoms, especially among patients with contamination symptoms.^12,13,15,20,35^ However, besides the contamination symptoms, responsibility for harm and unacceptable thoughts were among the deteriorated OC symptom dimensions.^36,37^ Only a few studies had examined OCD subtypes.^19,36,37,39^ There were also contradictory results showing that the pandemic had no impact on some fields of OCD.^45,46^ Because the complex nature and heterogeneity of OCD is known, variable responses to the COVID-19 outbreak may also be considered normal. Anxiety about COVID-19 was a part of OCD for some, while it is a separate issue for others. Although some OCD groups experienced high anxiety about COVID-19, a great majority reported that their primary problem was pre-existing OCD concerns, while few reported COVID-19 as their main concerns.^15,18,28^

Regarding OCD symptom dimensions, participants’ self-reports suggested that worsening of OC symptoms was more strongly associated with symptoms of contamination and responsibility for harm than with intrusive thoughts or symmetry symptoms.^12,36^ These results were in line with past research demonstrating contamination concerns about past pandemics.^48^

Interestingly, there were several studies showing a rise in OC symptom severity in adults despite the possibility of psychopharmacological treatment and psychotherapy.^12,24,25,28,35,36,37^ However, 3 different studies highlighted that the impact of COVID-19 on OCD patients was only minimal, and symptoms even showed improvement.^39,41,45^ One of these studies noted that pandemic may be an opportunity for exposure.^39^

It is important to underline that providing treatment can affect outcomes of the OC symptoms because in most studies, the method of treatment was uncertain or only some reported receiving treatment. Briefly, data about the method and course of treatment were uncertain and heterogeneous.

Regarding the measures used in data collection, studies preferred quantitative measures with psychometric properties and/or qualitative tools such as questionnaires. Y-BOCS had been used frequently, considering that it was the gold standard to measure the severity of OCD symptoms.^49^ Regarding self-report measures, the OCI-R was used.^50^ Most of the articles we analyzed preferred online methods such as phone interviews and online surveys to comply with COVID-19 precautions.

Heterogeneity of measurements may affect results; a face-to-face interview conducted by researchers may be more certain than phone interviews or self-report questionnaires. In addition, studies did not specify whether the participants had COVID-19. Obsessive-compulsive disorder patients affected by COVID-19 can be expected to experience worsening of their symptoms.

While responses have been variable, significant mental health problems appear for many adults with OCD because COVID-19 has an effect on worsening symptoms and complex therapy for many. Further studies are needed to identify the mental health burden of COVID-19 on OCD patients. In times of quarantine and social distancing, increasing accessibility to treatment and ensuring treatment delivery in a safe and effective way will be important at this unprecedented time.

Although studies reporting worsened OCD symptoms were significant, data on treatment results were scarce. Follow-up studies were few and factors such as sociodemographic characteristics, subtypes, comorbid conditions, treatment, and family support seem to have not been adequately studied. More studies are essential to understand how variables exacerbate OC symptoms in the OCD population.

## Conclusion

Results were conflicting. Although studies reported more information on worsening OCD symptoms, data on treatment were scarce. Studies evaluating follow-up results were few. Factors such as sociodemographic characteristics, subtypes, comorbid conditions, treatment, and support seem to have not been adequately studied.

Our results indicate the need for the development of the studies and seem important for clinicians and the scientific community in terms of shedding light on the impact of the COVID-19 pandemic on OCD, a psychiatric disorder that causes significant impairments of functionality. This information is essential to capitalize on more appropriate and early interventions and to understand how variables exacerbate OC symptoms in the OCD population.

## Figures and Tables

**Table 1.** Analysis of the Studies

References/Country	Purpose	Research Design	Sample Characteristics	Comorbidity	Treatment	Conclusion criteria	Monitoring Period	Main Results
Wheaton et al, USA^12^	Investigating how COVID-19 affects OCD patients.	Cross-sectional study, quantitative and qualitative evaluation	NP = 252 NC = 305	Previous diagnoses were unclear, and increased symptoms of depression and anxiety were detected in the study.	63.5% psychopharmacological treatment, 61.2% psychotherapy (CBT, CBT+ERP, supportive psychotherapy)	Online	April-December 2020	76.2% reported worsening OCD symptoms. They reported increased concern about COVID-19. 58.3% reported concern about COVID-19 as an obsession with OCD symptoms and 41.7% reported concern about COVID-19 as a separate issue. OCD symptoms were associated with contamination and harm liability. They reported that COVID-19 was affecting their treatment, but they were satisfied with the treatment providers.
Zheng et al, China^13^	Investigating the prevalence of OCD in an urban population during the COVID-19 pandemic, identifying clinical features and risks.	Cross-sectional study	N = 541	Depression and anxiety disorders	Undetermined	Online	9-19 July 2020	OCD prevalence was 17.96%. Aggression and contagion obsessions and compulsions to clean, check, and repeat were evident. Being single, being a student, family history of OCD and other mental disorders, comorbid psychiatric comorbidity, and prolonged sleep latency were risk factors for OCD.
Hojgaard et al, Denmark^14^	To evaluate self-reported changes in OCD symptom severity in adults with OCD during the COVID-19 pandemic and whether the COVID-19 pandemic could trigger self-reported symptoms of contamination in individuals with no history of OCD	Cross-sectional study	N = 201	Depression and anxiety disorders	Undetermined	Questionnaire via e-mail	6-29 April 2020	61.2% worsening, 10.4% improvement, and 28.4% unchanged. A group of 29.4% reported that they were more accepting of their obsessive thoughts. 64.7% reported symptoms related to contamination and 56.2% reported symptoms related to harm. Occurring during the non-previous pandemic process, there were only 5 participants reporting symptoms of contamination and 2 participants reporting symptoms related to harm.
Samuels et al, USA^15^	Evaluating whether COVID-19 measures trigger OCD.	Cross-sectional study	N = 2117	Depression and anxiety disorders		Online	September 2020	22% reported contagion obsessions and 20% phobia of contagion. The rate was higher in men. Age and symptoms were inversely related and other sociodemographic variables were also related. Depression, anxiety, doubt, and COVID-19 risk scores increased the likelihood of all outcomes.
Kavaladze et al, USA^16^	Evaluating how COVID-19 affects OCD.	Cross-sectional study	N = 196	Undetermined	Undetermined	Online	June-August 2020	92.9% worsening of OCD symptoms, but symptom characteristics were variable, symmetry and perception of unity were less altered in symptoms. 95.5% stated that having OCD made it difficult to cope with the pandemic.
Loosen et al, United Kingdom^17^	Evaluating the course of symptoms of OCD, depression, and anxiety during the first wave of the pandemic, and how seeking information and following guidelines regarding COVID-19 are affected by these symptoms.	Longitudinal study	N = 406	Depression and anxiety disorders	Undetermined	Online	April-August 2020	In the first wave of the pandemic, and OCD, depression and anxiety increased. Depression decreased in the next wave, anxiety flattened, OCD continued to increase. Information-seeking behavior increased at the beginning of the pandemic, then decreased, and was especially related to OC symptoms.
Acenowr ve Coles, USA^18^	Evaluation of intrusive thoughts about COVID-19 and OCD in individuals without OCD.	Cross-sectional study	N = 2020	Anxiety disorder	Undetermined	Online, face to face, telephone	February-April 2020	Individuals with high OCD symptoms reported having both OCD and COVID intrusions at a similar frequency to those with low OCD symptoms. OCD symptom severity was significantly correlated with the frequency of COVID intrusions and the amount of distress they cause. Distress from COVID-related intrusions was not significantly associated with OCD symptom severity.
Fontenelle et al, USA^19^	To evaluate whether OCD-related disorders are affected by the pandemic process.	Cross-sectional study	N = 829	Excluded	Undetermined	Online, face to face	April 2020	Significant aggravation of various OCD symptoms was seen in the general population, particularly OCD, HD, and SPD. There was a deterioration in quality of life and an increase in disability. Increased vulnerability to worsening symptoms may be related to certain sociodemographic and clinical characteristics, including gender, prior diagnosis and seeking treatment, specific OCD symptoms and compulsions, severity of schizotypal features, and amount of stress experienced in relation to the COVID-19 pandemic.
Jelinek et al, Germany^20^	To evaluate how COVID-19 affects OCD, particularly washing compulsions.	Cross-sectional study	N = 394	Depression	Undetermined	Online	March-May 2020	72% increase in OCD symptoms, further increase in washers
Jelinek et al, Germany^21^	To evaluate trajectories and associated factors of OCD in the COVID-19 process.	Longitudinal study	N = 1207	Undetermined	Undetermined	Online	Two different evaluations were in March 2020 (t1) and June 2020 (t2). t1; COVID-19 quarantine has just begun, t2; quarantine measures have been relaxed.	66% asymptomatic (OCD−/OCD−), 18% continuous symptomatic (OCD+/OCD+), 10% delayed onset (OCD−/OCD+), 6% recovery (OCD+/OCD−)
Taher et al, Iraq^22^	Evaluation of OCD prevalence, related factors, and correlations among medical students.	Cross-sectional study	N = 1664	Anxiety	No treatment	Online	August-October 2020	57% were evaluated as normal and 43% were evaluated as having probable OCD requiring further evaluation. 70% had accompanying psychiatric symptoms, anxiety, and stress. Unpleasant thoughts were the most common symptoms with 51.8%. Surprisingly, washing and contamination scales were low at 14% and 19.4%, respectively, while recurrences of certain numbers were minimal at 8%. The presence of other psychological symptoms such as anxiety, depression, sleep disorders, feeding disorders, and stress were significantly associated with probable OCD. The rate of probable OCD was higher in those who were earlier medical students.
Wheaton et al, USA^23^	Assessing the relationship of anxiety about COVID-19 with intolerance to uncertainty and OCD and health anxiety	Cross-sectional study	N = 720	Anxiety	Undetermined	Online	March 2020	OCD symptoms and health anxiety were associated with fear of COVID-19 spreading. Intolerance of uncertainty was responsible for some of the association between OCD symptoms, health anxiety, and fear of COVID-19.
Liao et al, China^24^	To evaluate the immediate and long-term effects of the COVID-19 pandemic on patients with obsessive- compulsive disorder	Longitudinal study, 1-year follow-up study	N = 110, 1-year follow-up of 64 patients	Depression and anxiety disorders	55.45% received drug treatment and 39.09% received clinical follow-up.	Online survey and clinical interviews	February 26-March 25, 2020-February 26-March 25, 2021	OCD, depressive and anxiety symptoms showed worsening during early COVID-19 and the adverse effect persisted at 1-year follow-up. Female gender, anxiety about COVID-19, and baseline OCD symptom severity are risk factors for exacerbation of OCD symptoms during early COVID-19, while optimism as a mental resilience factor is a protective factor against exacerbation of OCD.
Storch et al. USA^25^	To report the clinicians’ assessment of the impact of the COVID-19 pandemic on exposure and response prevention outcomes in adults and adolescents with obsessive-compulsive disorder.	Cross-sectional study	N = 232	Undetermined	ERP	The online questionnaire was sent electronically via e-mail to 595 clinicians who regularly provide CBT to adults and children with OCD and anxiety. 137 clinicians completed questionnaires for at least one client.		The findings suggest that the COVID-19 pandemic is associated with attenuation of ERP progression from expected rates in most patients during the first few months of the pandemic; clinicians showed that 38% of their patients’ symptoms worsened during the pandemic and that although 47% participated in ERP, symptoms did not change.
Al-Shatanawi et al. Jordan^26^	Screening of self-reported obsessions to COVID-19 preventive measures among undergraduate medical students in the early stage of the pandemic	Cross-sectional study	N = 1404	Anxiety	No treatment	Online	March 2020	Self-reported obsession levels about COVID-19 measures were found to be higher in female students and those who attended trainings related to COVID-19. There was no difference between universities and academic levels. It showed that 6.8% of the participants reported obsessions about preventive measures against COVID-19, while 93.2% (n = 1,308) had no obsessions.
Ojalehto et al. USA^27^	To evaluate the relationship of COVID-19 anxiety with obsessive-compulsive symptom dimensions, anxiety sensitivity, and bodily sensations.	Cross-sectional study	N = 438	Depression and anxiety disorders	No treatment	Online	August-November 2020	Obsessive-compulsive symptoms related to contamination, fear of arousal-related body sensations (anxiety sensitivity), and body alertness each predicted more severe anxiety related to the pandemic. Obsessive-compulsive symptoms related to the responsibility to cause harm also emerged as a predictor.
Alonso et al. Spain^28^	To evaluate the impact of the COVID-19 pandemic on OCD patients.	Case–control study	Number of patients (NP) = 127 Number of controls (NC) = 237	Depression and anxiety disorders	Psychopharmacological treatment	Telephone	April-May 2020	The rate of contracting COVID-19 was the same between the OCD and control groups. The OCD group scored higher in perceived anxiety and depression levels, suicidal thoughts and experienced more changes in eating and sleeping patterns. Depressive symptoms were more common in the OCD group, while the control group identified more pathological behaviors related to impulse control, including pathological gambling, compulsive internet use, or compulsive buying. OCD patients tended to do less physical exercise and yoga and reported that it was more difficult for them to establish a daily routine. Both groups used online or face-to-face meetings as coping strategies for coping with emotional distress.
Ioana Mesterelu et al, Romania^29^	Obsessive-compulsive symptoms and responses to COVID-19	Cross-sectional study	N_first evaluation_=159 N_second evalauation_=56			Online evaluation via social media announcement	March-April 2020 November-December 2020	It showed that OCD symptom levels significantly predicted anxiety about COVID-19, adaptive and maladaptive behaviors. OCD symptom levels at the onset of the pandemic proved unpredictable later anxiety to adaptive or maladaptive behavior related to COVID-19.
Ji et al. China^30^	The effect of the COVID-19 pandemic on obsessive-compulsive symptoms among university students	Longitudinal study	N_questionnaire1_=13478 N_questionnaire 2=8162_ N_questionnairet 3= 8511_	Anxiety	No treatment	Online	Survey 1 was conducted on February 8, 2020, after a 2-week quarantine period without classes; survey 2 was conducted on March 25, 2020, where participants took online courses for 2 weeks; and survey 3 was conducted on April 28, 2020, with no new cases reported for 2 weeks.	In questionnaire 1, 11.3% of the participants were identified as having probable OCD. In surveys 2 and 3, 3.6% and 3.5% of respondents had scores indicating probable OCD, respectively. Those with higher fear intensity, higher anxiety level, male gender, sibling(s), and specializing in a non-medical discipline were more likely to have a higher Y-BOCS score on all questionnaires.
Abba-Aji et al. Canada^31^	To assess the prevalence of new-onset obsessive-compulsive symptoms in a Canadian province during the COVID-19 pandemic.	Cross-sectional study	N = 6041	Depression and anxiety disorders	No treatment	Online	March 2020	The prevalence of OCD symptoms increased during the COVID-19 pandemic at a significantly higher rate than reported pre-pandemic rates for the sample population. Presenting with OCD symptoms increased the likelihood of presenting with high stress, possibly GAD, and possibly MDD.
Berman et al, USA^32^	To examine the association between COVID-19 impact and OCD symptoms on college students	Cross-sectional study	N = 840	Undetermined	Undetermined	Online questionnaire	October-December 2020	There was a significant relationship between COVID-19 impact and OC severity and highlighted that emotion regulation difficulties may help explain this association.
Al-Hassani and Mufaddel, United ArabEmirates^33^	To investigate the prevalence of obsessive-compulsive disorder (OCD) COVID-19 pandemic among the general population of the United Arab Emirates.	Cross-sectional study	N = 702; M = 371, F = 325	Generalized anxiety (27.3%), phobia (1.8%), depression (19.1%), bipolar mood disorder (1.8%), OCD (6.3%), and panic attacks (8.2%).	No treatment	Online	October 2020-January 2021	The study found increased prevalence of OCD during the COVID-19 pandemic, which is significantly higher among people with positive COVID-19 infection compared to those with negative COVID-19 test results.
Mahaffey et al. USA^34^	To examine the prevalence of OC symptoms in pregnant women in the USA	Cross-sectional study	4451 pregnant women	Undetermined	No treatment	Online	April-May 2020	Clinically significant OC symptoms were present in 7.12% of participants, more than twice as high as rates of peripartum OCD reported prior to the pandemic. Younger maternal age, income loss, and suspected SARS-CoV-2 infection were all associated with higher OC symptoms.
Davide et al, Italy^35^	To investigate how COVID-19 affects the symptoms of patients with OCD. The post-epidemic status of those who were in remission before the epidemic and those who were not evaluated.	Cross-sectional study	NP = 30 M = 14 F = 16	Comorbidity in 5 people (3 personality disorders + 2 mood disorders)	Psychopharmacological treatment/ CBT+ psychopharmacological treatment	Face to face interview	3 weeks before quarantine—just after quarantine	They reported worsening OCD symptoms, particularly contamination-related symptoms. Worsening was more common in those who were in remission before quarantine.
Khosravani et al, Iran^36^	To evaluate changes in OCD symptom dimensions and severity during the pandemic in a population with a previous diagnosis of OCD.	Cross-sectional study	N = 270	Depression and anxiety disorders	psychopharmacological treatment +CBT	Face to face interview	May-July 2020	Increase in all symptoms and OCD severity, not only contamination but also harm responsibility, symmetry, and unacceptable thoughts are affected.
Khosravani et al, Iran^37^	To evaluate the relationship between COVID-19 stress response, suicide risk, and coping attitudes of OCD patients.	Cross-sectional study	N = 304	Depression and anxiety disorders, bipolar disorder, and substance abuse	Psychopharmacological treatment and psychotherapy	Face to face interview	June-October 2020	Responsibility to harm and unacceptable thoughts, as well as OCD severity, stress response to COVID-19, traumatic stress response control compulsion, comorbid depression and anxiety, and lifetime suicide attempts affect suicidal ideation.
Benatti et al, Italy^38^	Evaluating how COVID-19 affects OCD patients.	Cross-sectional study	N = 123	Undetermined	Undetermined	Telephone and face to face interview	June 2020	1/3 reported worsening of OCD symptoms. Those who reported obsessive worsening and those who were not compared. Mostly harm/violence and multiple obsessions and washing/cleaning compulsions were increased. More drug compliance, suicidal ideation, internet control for reassurance, need for family support, and sleep disorders were reported more in those with obsessive worsening.
Kuckertz et al, USA^39^	Evaluation of the data of OCD patients whose treatment continues during the COVID-19 process.	Longitudinal study, case series report	N = 8	Undetermined	ACT–ERP therapy	Face to face follow-up	April-May 2020	Improvement has been reported in OCD patients with ACT–ERP therapies.
Tandt et al, Belgium^40^	Evaluating the importance of family support for OCD patients qualitatively.	Cross-sectional qualitative study	N = 22 patient N = 13 family	Psychotic disorders and substance abuse excluded	Undetermined	Face to face interview	April 2020	Almost all participants reported that the COVID-19 crisis led to an increase in OCD symptoms, a decrease in the ability to use coping strategies, and families tended to respond with increased FA. The importance of including family support in the therapy process highlighted
Carmi et al, Israel^41^	To evaluate the results of 2-month and 6-month treatment impressions of OCD patients.	Longitudinal study	N = 113, 2-month follow-up N = 90 6-month follow-up	Undetermined	CBT, ERP, and pharmacological treatment	Face to face follow-up	April-May and September 2020	No exacerbation of symptoms was observed in 84% at 2-month follow-up and 96% at 6-month follow-up.
Yassa et al, Turkey^42^	Evaluation of anxiety levels and OCD symptoms of pregnant and non-pregnant women during the COVID-19 pandemic period.	Case–control study	N = 203 pregnant N = 101 non-pregnant	Anxiety	Undetermined	Face to face interview	April 2020	Pregnant women showed more OCD and less anxiety symptoms than non-pregnant women. State anxiety levels did not differ between the trimesters. Anxiety was found to be positively associated with all obsessive-compulsive symptoms except the cleanliness and hygiene subscale.
Ergenc et al, Turkey^43^	Evaluation of OCD, depression, and anxiety in healthcare workers working in the COVID-19 service and other services.	Case–control study	N = 198 NC = 130 NS = 68	Depression and anxiety disorders	Undetermined	Face to face interview	April 2020	Health workers working in the COVID-19 service showed a significant increase in OCD, depression and anxiety symptoms compared to healthcare workers working in other services. Sociodemographic characteristics of the participants were similar in both groups.
Tukel et al, Turkey^44^	To investigate how the pandemic has affected OCD patients and the relationship between the clinical features and the fear and obsession with COVID-19.	Cross-sectional	N = 60 P = 30 HC = 30	At least 1 psychiatric disorder comorbidity was determined in 66.7% of the patients. Major depression (30%) and specific phobias (30%) were the most common comorbid	Pharmacological treatment	Face to face interview	September 2020 -January 2021	Obsessive-compulsive symptom severity worsened in 60% of OCD patients during the pandemic. COVID-19 obsession levels were higher in OCD patients than in healthy controls. COVID-19 fear levels did not differ between the OCD and healthy control groups. COVID-19 obsession levels were correlated with anxiety severity in OCD and healthy control groups.
Chakraborty et al, Iran^45^	To evaluate whether the symptoms of OCD patients increase during the COVID-19 period.	Cross-sectional study	N = 84	Undetermined	57 of the 84 patients took their medications regularly, 13 of them irregularly because of the fear of drug shortage if they could not find the drug, and 14 of them stopped taking due to not being able to find them in nearby pharmacies.	Telephone	April-May 2020	Only 5(6) patients reported exacerbation of symptoms. All 5 patients were not taking their medication because they could not go to the pharmacy. All other patients reported that their symptoms were the same as before.
Alcazar et al, Spain^46^	Evaluating coping strategies of OCD patients during COVID-19 pandemic process.	Case–control study	N = 122 patient N = 115 control	Depression and anxiety disorders	67% of OCD patients received pharmacological treatment (antidepressant = 69.50%, antipsychotic+antidepressant = 30.50%) and 100% received psychological treatment.		April 2020	Depression and anxiety symptoms were higher in the OCD group. Positive reframing, acceptance, and humor were higher in the control group. The OCD group showed better results on instrumental support and religion. The OCD group showed higher rates of denial and self-blame, and cognitive coping and spiritual coping were statistically significant. While the control group mostly used cognitive strategies, the OCD group mostly used emotional strategies.
Cox et al, USA^47^	Evaluating whether insomnia before COVID-19 would be a predictor for OCD symptoms	Cross-sectional study	N = 369	Undetermined	Undetermined	Face to face interview	April 2020	There was a small increase in OCD symptoms after the pandemic compared to pre-COVID-19. Having a pre-pandemic insomnia symptom resulted in a small increase in OCD symptoms in the post-COVID era.

ACT, acceptance and commitment therapy; ERP, exposure response prevention; HD, hoarding disorder; OCD, obsessive-compulsive disorder; SPD, skin-picking disorder.
